# Milk Ingredients in Meat Products: Can Autoclaving and In Vitro Gastroduodenal Digestion Mitigate Their IgE-Binding Capacity?

**DOI:** 10.3390/nu13030931

**Published:** 2021-03-13

**Authors:** Caterina Villa, Simona L. Bavaro, Elisabetta De Angelis, Rosa Pilolli, Joana Costa, Simona Barni, Elio Novembre, Isabel Mafra, Linda Monaci

**Affiliations:** 1 REQUIMTE-LAQV, Faculdade de Farmácia, Universidade do Porto, Rua de Jorge Viterbo Ferreira, 228, 4050-313 Porto, Portugal; caterinavilla@hotmail.com (C.V.); jbcosta@ff.up.pt (J.C.); isabel.mafra@ff.up.pt (I.M.); 2 ISPA-CNR, Institute of Sciences of Food Production of National Research Council of Italy, Via Amendola 22/O, 70126 Bari, Italy; simona.bavaro@ispa.cnr.it (S.L.B.); elisabetta.deangelis@ispa.cnr.it (E.D.A.); rosa.pilolli@ispa.cnr.it (R.P.); 3 Allergy Unit, Department of Pediatrics, Meyer Children’s University Hospital, 50139 Florence, Italy; simona.barni@meyer.it (S.B.); elio.novembre@unifi.it (E.N.)

**Keywords:** milk allergen, autoclaving, meat products, simulated digestion, IgE reactivity, LC-MS/MS, hypoallergenic product

## Abstract

The food industry commonly uses milk ingredients as technological aids in an uncounted number of products. On the other hand, milk contains allergenic proteins causing adverse allergic reactions in sensitized/allergic individuals. This work intends to evaluate the effect of autoclaving and in vitro digestion on the allergenicity of milk proteins incurred in meat products. Protein profiles of raw and autoclaved sausages without and with the addition of 10% of milk protein concentrates were analyzed by gel electrophoresis and liquid chromatography–mass spectrometry. Additionally, residual IgE-reactivity was evaluated by immunoblot analysis using pooled sera of cow’s-milk-allergic individuals followed by bioinformatic analysis. Results showed that autoclaving led to an increase in protein fragmentation (higher number of short peptides) and consequently to a higher digestion rate, that was found to be more pronounced in β-casein. The IgE-binding capacity of milk proteins seems to be reduced after autoclaving prior to digestion, with a residual reactivity in caseins, but was eliminated following digestion. This study highlights the importance of autoclaving as a processing strategy to produce hypoallergenic formulas.

## 1. Introduction

Cow’s milk proteins are often used as ingredients in the food industry due to their technological/functional characteristics, aiming at improving the aspect, taste, and texture of foodstuffs, as well as their nutritional value [1]. However, milk proteins are common food allergens, responsible for one of the most recurrent food allergies in early childhood, with a prevalence between 0.6% and 3% in children under the age of 6 years [2]. Due to the high number of food products containing milk ingredients, accidental exposure to milk proteins is very common, representing a constant threat to allergic individuals. Those patients are then forced to carry out an elimination diet in order to avoid the occurrence of mild to severe allergic reactions, such as cutaneous, respiratory or gastrointestinal reactions, and even life-threatening occurrences such as systemic anaphylaxis [2,3].

Cow’s milk proteins are divided in two major groups according to their solubility at pH 4.6 and 20 °C: caseins (αS_1_-, αS_2_-, β-, κ-caseins) and whey proteins (α-lactalbumin (α-LA), β-lactoglobulin (ß-LG), bovine serum albumin (BSA), lactoferrin, and immunoglobulins (Ig)), accounting for 80% and 20% of the total protein content, respectively [2]. According to the World Health Organization and International Union of Immunological Societies’ (WHO/IUIS) list of allergens, cow’s milk allergens are classified as: Bos d 8 (a name that designates all caseins) or Bos d 9 (αS_1_-casein), Bos d 10 (αS_2_-casein), Bos d 11 (β-casein), Bos d 12 (κ-casein) for each individual casein; Bos d 4 (α-LA); Bos d 5 (ß-LG); Bos d 6 (BSA); and Bos d 7 (Ig) [4]. Considering that more than 50% of sera from milk-allergic patients reacts with caseins, α-LA, ß-LG, and BSA, these proteins are considered major allergens in cow’s milk, while Ig is classified as a minor allergen [2].

Milk and milk proteins can be found in numerous food matrices, being exposed to distinct types of processing, until they are presented to consumers as final products. Food processing, both conventional methods (e.g., heat treatment or fermentation) and novel methods (e.g., high pressure, irradiation, or ultrasound) can induce chemical and physical changes of milk proteins, differently affecting their allergenicity [2,5]. These alterations may induce the destruction of conformational epitopes, either by denaturation, by aggregation phenomena, or by the development of chemical reactions among the different food matrix components (proteins, fat, and sugars), restricting the accessibility of the protein to the immune system and, therefore, causing a reduction in the allergic response [6,7]. On the other hand, the formation of neo-epitopes and/or a decrease in protein digestibility due to protein–matrix interactions might potentially increase the IgE-binding capacity of proteins [8]. The large amount of proteins in a food matrix may improve the resistance against gastrointestinal degradation and originate a competitive environment for enzyme cleavage, thereby postponing gastrointestinal proteolysis of food allergens [9].

The effects of food processing and food matrix on milk proteins have been extensively studied in order to control milk allergy [2]. Different strategies such as microwave [10,11], fermentation [12], high pressure [13], pulsed light [14], and ultra-sound [15] have been recently explored for their potential to modify the intrinsic allergenicity of milk proteins with interesting results. A study developed by Nowak-Wegrzyn et al. [16] demonstrated that about 70% of tested children were capable of ingesting milk in baked muffins without any immediate clinical symptoms. Bavaro et al. [17] compared the IgE-binding capacity of milk baked in the oven with baked milk within a muffin matrix, proving that interactions between milk proteins and food constituents during oven-heating can induce a possible reduction of milk allergenicity. Resistance to gastrointestinal digestion is also an important parameter to take into account since the potential of an allergen to trigger an immunoreaction results from the preservation of its structural integrity during the digestion process [18]. Several works using in vitro digestion models have been performed to study milk protein digestibility, but without the inclusion of the allergen in a food matrix, they lack the subsequent evaluation of the effect of the interactions between milk proteins and other food components [19,20,21,22,23,24]. These interactions should be carefully considered because they might greatly alter the structural properties of proteins as well as their IgE-binding and final allergenicity.

This work aimed at studying the effect of autoclaving on the IgE-binding capacity of milk ingredients used as technological aids in meat products, in combination with in vitro simulated gastroduodenal (GD) digestion. Proteomic profiles of digested model mixtures of turkey sausage samples spiked with milk protein concentrates (MPCs) were characterized before and after autoclaving treatment, and the IgE-binding capacity was tested by immunoblotting experiments with human sera of cow’s-milk-allergic patients (pooled sera) and bioinformatic search.

## 2. Materials and Methods

### 2.1. Chemicals

Bovine serum albumin (BSA), ammonium bicarbonate (AMBIC), iodoacetamide (IAA), dithiothreitol (DTT), along with chemicals for electrophoresis, namely sodium dodecyl sulfate (SDS), glycine, glycerol, and Coomassie Brilliant Blue-G 250 and purified proteins (β-LG, total caseins, α- and β-caseins) were provided by Sigma Aldrich (Milan, Italy). Acetonitrile (gold HPLC ultragradient), trifluoroacetic acid (TFA), and bromophenol blue were purchased from Carlo Erba Reagents (Cornaredo, Milan, Italy), while ultrapure water was produced by a Millipore Milli-Q system (Millipore, Bedford, MA, USA). Formic acid (MS grade) was purchased from Fluka (Milan, Italy), whilst 0.45 µm filters in polytetrafluoroethylene (PTFE) and 5 µm filters in cellulose acetate (CA) were purchased from Sartorius (Gottingen, Germany). Trypsin (proteomic grade) for the in-gel protein digestion was purchased from Promega (Milan, Italy). As for in vitro digestion experiments, potassium chloride (KCl), potassium dihydrogen phosphate (KH_2_PO_4_), sodium bicarbonate (NaHCO_3_), sodium chloride (NaCl), magnesium chloride hexahydrate (MgCl_2_(H_2_O)_6_), ammonium carbonate ((NH_4_)_2_CO_3_), sodium hydroxide (NaOH), hydrochloric acid (HCl), and calcium chloride (CaCl_2_) along with other analytical grade chemicals and enzymes (salivary α-amylase, pepsin, trypsin, chymotrypsin, pancreatic α-amylase, pancreatic lipase plus phospholipid, bile, serine protease inhibitor (PMSF = methyl-phenyl-sulfonyl fluoride)) were obtained from Sigma-Aldrich (Milan, Italy).

### 2.2. Production of Milk-Free and Incurred Model Sausages

The model samples used in this work were prepared following the recipe of industrial sausages incurred with 10% of MPC, a commercial product purchased by Formulab (Maia, Portugal), commonly used as a technological aid in the manufacture of hams and sausages. The product came with a detail sheet of specifications on chemical, physical, and microbiological parameters, thus no further characterization of the formulation was required. In addition, it is known that these types of concentrates contain caseins and whey proteins in the same quantity as whole milk (80% and 20%, respectively) [25,26]. For confirmation, only the protein content was checked by Kjeldahl protocol, obtaining a content of 83.4%. 

The mixture used for sausage preparation was made by the addition of 44% of minced turkey meat, 33% of pork fat, 2% of salt, and 21% of crushed ice, ground and homogenized in a laboratory knife mill (Grindomix GM200, Retsch, Haan, Germany) with 10 mL of a sterile phosphate-buffered saline solution (136 mM NaCl, 1.4 mM KH_2_PO_4_, 8.09 mM Na_2_HPO_4_·12H_2_O, and 2.6 mM KCl, pH 7.2) to facilitate homogenization. This raw mixture was used as a negative control (0% of MCP addition). The incurred model sausage containing 10% of MPC was prepared by the addition of 25.2 g of MPC in 184.8 g of the raw mixture taking into account the protein content of MPC measured by Kjeldahl. Model sausages with 0% and 10% of MPC were divided in two subsets each, one to be used as raw material and the other one submitted to an autoclaving processing (121 °C, 15 min, 1 bar) simulating the industrial production of sausages. In summary, the analyzed model sausages were as follows:

(a) raw model sausage with 0% MPC (SMPC0); (b) raw model sausage with 10% MPC (SMPC10); (c) autoclaved model sausage with 0% MPC (PSMPC0); and (d) autoclaved model sausage with 10% MPC (PSMPC10).

All model sausages were directly stored at −20 °C until further analysis.

### 2.3. Sera of Milk-Allergic Patients

Sera were obtained from seven milk-allergic children according to ethical requirements. Tests were conducted in accordance with the Declaration of Helsinki and all procedures of the study were approved by the local ethics committee (code 2018/128). Permission to participate in the study of all children was obtained and the written informed consent was signed by the parents. The allergy symptoms in general ranged from vomit, cough, and rhinitis to anaphylaxis. The clinical features of the allergic individuals enrolled in this study are reported in Table 1. Diagnosis of IgE-mediated allergy to cow’s milk was previously confirmed by positive skin prick test (SPT) and serum-specific IgE (ImmunoCAP, Phadia, Uppsala, Sweden) to cow’s milk and cow’s milk proteins (sIgE to cow’s milk, β-LG, α-LA, caseins). All sera were stored at −80 °C until further use.

### 2.4. Simulated In Vitro Gastroduodenal Digestion of Model Sausages

#### 2.4.1. Preparation of Gastroduodenal Fluid Stock Solutions 

In vitro GD digestion experiments were fulfilled according to a standardized static protocol, simulating chewing, gastric, and intestinal compartments as described by Minekus et al. [27]. Simulated salivary fluid (SSF, pH 7) was prepared in order to include KCl (15.1 mM), KH_2_PO_4_ (3.7 mM), NaHCO_3_ (13.6 mM), MgCl_2_(H_2_O)_6_ (0.15 mM), (NH_4_)_2_CO_3_ (0.06 mM), and HCl (1.1 mM) for pH adjustment. Simulated gastric fluid (SGF, pH 3) was prepared with KCl (6.9 mM), KH_2_PO_4_ (0.9 mM), NaHCO_3_ (25 mM), NaCl (47.2 mM), MgCl_2_(H_2_O)_6_ (0.1 mM), (NH_4_)_2_CO_3_ (0.5 mM), and HCl (15.6 mM) for pH adjustment. Simulated intestinal fluid (SIF, pH 7) was prepared with the addition of KCl (6.8 mM), KH_2_PO_4_ (0.8 mM), NaHCO_3_ (85 mM), NaCl (38.4 mM), MgCl_2_(H_2_O)_6_ (0.33 mM), and HCl (8.4 mM) for pH adjustment. CaCl_2_(H_2_O)_2_ (0.3 M) was also used, but it was not added to the electrolyte stock solutions as precipitation may occur.

#### 2.4.2. Assessment of Protein Solubility in Gastroduodenal Fluids before Enzymatic Digestion

Raw and processed model sausages without and with MPC addition (SMPC0, SMPC10, PSMPC0, PSMPC10) were treated sequentially with salivary fluids (SSF; 2 min), gastric fluid (SGF, 2 h), and duodenal fluid (SIF, 2 h), as described below. All steps were carried out in a shaking incubator (170 rpm, IKA^®^ KS 4000 i control) at 37 °C. Briefly, 1 g of each model sausage was mixed with 995 μL of SSF and 5 μL of CaCl_2_(H_2_O)_2_ (0.3 M) and incubated for 2 min to simulate chew conditions. Then, the obtained mixture was mixed in a 1:1 proportion with SGF and 1 μL of CaCl_2_(H_2_O)_2_ (0.3 M) and the pH adjusted to 3.0 for mimicking the gastric environment. The final volume of 4 mL was completed with ultrapure water (Millipore MilliQ system, Bedford, MA, USA). The final mixture was then left shaking at 37 °C for 2 h. Afterward, 1:1 parts of SIF and 8 μL of CaCl_2_(H_2_O)_2_ (0.3 M) were added to gastric mixture, the pH was adjusted to 7.0 and a final volume of 8 mL was completed by adding MilliQ water in order to simulate duodenal conditions. A last incubation during 2 h at 37 °C was performed. The final mixture was then centrifuged for 15 min at 17,000× *g* at 4 °C (refrigerated micro-centrifuge D3024R, Scilogex) and the supernatant was collected and filtered through a 5 μm cellulose acetate syringe filter. The concentration of total solubilized proteins was assessed by Bradford assay (Quick Start™ Bradford Protein Assay, Bio-Rad Laboratories, Segrate, MI, Italy) according to the manufacturer’s guidelines (standard protocol with a 1 mL cuvette assay and 5 min incubation time). Bovine serum albumin was used as the reference protein. Mean values of three independent samples were calculated and standard deviation was reported as error bars in the histograms. All mixtures were stored at −20 °C until use and filtered through 0.45 μm PTFE filters just before electrophoretic analysis.

#### 2.4.3. In Vitro Gastroduodenal Digestion of Model Sausages

Raw and autoclaved model sausages without (0%) and with (10%) MPC addition were submitted to in vitro GD digestion according to the standardized protocol described by Minekus et al. [27]. All steps were performed in a shaking incubator at 37 °C, at 170 rpm. Oral phase was simulated by adding 620 μL of SSF and 5 μL of CaCl_2_(H_2_O)_2_ (0.3 M) with the addition of 75 U/mL of human salivary amylase (Sigma-Aldrich, St. Louis, MO, USA) to 1 g of model sausage, which was then kept incubating for 2 min. Afterward, the chewed mixture was mixed in a 1:1 proportion with SGF and 1 μL of CaCl_2_(H_2_O)_2_ (0.3 M) containing 2000 U/mL of gastric pepsin (Sigma-Aldrich) and 0.17 mM of phospholipids (Sigma-Aldrich) to mimic gastric digestion. The pH was adjusted to 3.0 with HCl and a final volume of 4 mL was completed with ultrapure water. Mixtures were then incubated for 2 h at 37 °C. For the duodenal phase, a last incubation at 37 °C for 2 h was carried out after incorporating of SIF (1:1 *w*/*v*) and 8 μL of CaCl_2_(H_2_O)_2_ (0.3 M) with 100 U/mL of pancreatin (Sigma-Aldrich) and 10 mM of bile salts (Sigma-Aldrich) into the chyme. The pH was adjusted to 7.0 with NaOH and a final volume of 8 mL was completed by adding MilliQ water. Phenyl methane sulfonyl fluoride (PMSF) was added to stop the enzymatic reaction. A centrifugation at 17,000× *g* during 15 min (4 °C) was then performed, and the supernatant was collected and stored at −20 °C until further analysis. The total protein concentration was calculated by Bradford assay as described in Section 2.4.2. All mixtures were filtrated through 5 μm cellulose acetate syringe filters just before electrophoretic analysis.

### 2.5. SDS-PAGE Analysis

Sodium dodecyl sulfate-polyacrylamide gel electrophoresis (SDS-PAGE) was performed on 8–16% polyacrylamide pre-cast gels (8.6 cm × 6.7 cm × 1 mm) using a Mini-Protean Tetra Cell equipment (Bio-Rad Laboratories, Segrate, MI, Italy). Protein digests (10 µg) obtained in Section 2.4.2 and Section 2.4.3 were separated under reducing conditions. Protein solutions were mixed with Laemmli buffer [28] (62.5 mM TrisHCl, pH 6.8, 25% glycerol, 2% SDS, 0.01% bromophenol blue, 100 mM DTT) in a 1:1 proportion and then denatured for 5 min at 95 °C. The electrophoretic separation was performed in a running buffer (25 mM Tris, 192 mM glycine, 0.1% SDS) at 100 V until the end. Gels were stained by a Coomassie Brilliant Blue G-250 solution. The bands were detected on a ChemiDoc™ Imaging System (Bio-Rad Laboratories, Segrate, MI, Italy). Precision Plus Protein™ All Blue Standards (10–250 kDa, Bio-Rad Laboratories, Segrate, MI, Italy) were used as protein molecular weight references.

### 2.6. Immunoblot for IgE-Binding Assay

SDS-PAGE of protein solution of undigested and enzymatically digested sausages (corresponding to 10 µg of proteins loaded for SMPC0, SMPC10, PSMPC0, and PSMPC10) were electroblotted onto a 0.2 µm nitrocellulose membrane (Bio-Rad Laboratories, Segrate, MI, Italy) using a Trans-Blot Cell (Bio-Rad Laboratories, Segrate, MI, Italy) for 7 min (1.3 A, 25 V). Immunoblotting tests were fulfilled according to the protocol reported by Bavaro et al., 2019 [17]. As primary antibody, the pooled sera of a total of seven allergic children previously diluted in TBS-T (pH 7.4, 10 mM Tris, 50 mM NaCl, 0.1% Tween 20) at a 1/50 ratio was used and kept shaking overnight at 4 °C, while as a secondary antibody a goat anti-human IgG (H + L) horseradish peroxidase (HRP) Conjugate (Bio-Rad Laboratories) diluted 1/5000 (*v*/*v*) in TBS-T was added. Final images were obtained on a ChemiDoc^TM^ MP Imaging System.

### 2.7. LC-MS/MS Analysis

#### 2.7.1. Purification of Gastroduodenal Digested Samples

GD protein digests obtained in Section 2.4 were purified by Sep-Pak C18 cartridges (50 mg, 1 mL, Waters spa, Milan, Italy) according to the following protocol: (i) conditioning/equilibration step of column with methanol (3 × 1 mL) and SIF (3 × 1 mL), (ii) loading of digested sample (1 mL), (iii) washing step (0.5 mL of MilliQ water), (iv) elution step (1 mL H_2_O:CH_3_CN 10:90 + 0.1% formic acid). Finally, the eluates were filtrated through 0.2 µm PTFE filters, and 20 µL was injected into the LC-MS apparatus.

#### 2.7.2. In-Gel Protein Digestion

Selected protein bands from the SDS-PAGE were cut and submitted to in-gel digestion procedure according to De Angelis et al. [29]. Each sample was resuspended in 70 μL of H_2_O/acetonitrile, 90/10 + 0.1% formic acid (*v*/*v*), and 20 µL was further injected into the LC/MS apparatus.

#### 2.7.3. Protein Identification by Untargeted LC-MS/MS Analysis

Digests of model sausages and selected protein bands from SDS-PAGE analysis were analyzed by using a Q-Exactive™ Plus Hybrid Quadrupole-Orbitrap™ mass spectrometer coupled to an ultra-high-performance liquid chromatography (UHPLC) pump systems (Thermo Fisher Scientific, San José, CA, USA). Peptide mixture was separated on a reversed-phase Aeris peptide analytical column (internal diameter 2.1 mm, length 150 mm, particle size 3.6 μm, porosity 100 Å, Phenomenex, Torrance, CA, US) at a flow rate of 200 μL/mL according to the following conditions: from 0 to 50 min solvent B increased from 10% to 55%, at 50 min stepwise from 55% to 85%, then kept constant for 15 min, at 65 min down to a constant 10% during 20 min for column conditioning before following injection. As mobile phases, the solution H_2_O + 0.1% of formic acid (A) and acetonitrile + 0.1% of formic acid (B) were employed. MS spectra were acquired in the mass range of 200–2000 m/z by running the instrument in data dependent (FullMS-dd2) acquisition mode and only positive ions were considered in this study. Other MS parameters were the same as described in Bavaro et al. [27]. Final MS raw data were processed via the commercial software Proteome Discoverer™ version 2.1 (Thermo-Fisher-Scientific, San José, CA, USA) and protein identification was achieved by Sequest HT searching against a pig-, turkey-, and cow-customized database extracted by UniProt DB basing on the taxonomy codes of *Sus scrofa* (ID: 9823), *Meleagris gallopavo* (ID: 9103), and *Bos taurus* (ID:9913). The sequences of digestive enzymes used for simulating GD digestion were included as well. The identification of tryptic peptides originated by in-gel digestion experiments was fulfilled by setting the mass tolerance on the precursor and fragment ions at 5 ppm and 0.05 Da, respectively. A minimum of two peptide-spectrum matches with confidence at least medium (false discovery rate (FDR) < 5%), were assessed for protein identification.

### 2.8. Bioinformatic Analysis of the Residual Immunoreactivity of Milk-Enriched Raw and Processed Sausages after Gastroduodenal Digestion

Peptide sequences identified in digests of raw and processed model sausages with MPCs were searched in the Immune Epitope database (IEDB) (https://www.iedb.org/, accessed on 15 September 2020) in order to screen epitope linear sequences resisting to GD digestion. The IEDB results were filtered as follows: linear sequence for epitope structure, substring or exact match for BLAST option, human as host, and allergic reaction as disease.

## 3. Results and Discussion

### 3.1. Effect of Autoclaving on the Solubility and Enzymatic Digestion of Milk Proteins in Model Sausages under Simulated Gastroduodenal Conditions

#### 3.1.1. Assessment of Protein Solubility

As a first step, the impact of autoclaving on the solubility of endogenous (turkey and pork) and exogenous (milk) proteins of model sausages under investigation was assessed by estimating protein content with a colorimetric kit based on the Bradford assay, as previously reported [30,31,32]. Specifically, the assays were carried out on the protein fraction solubilized into the biological fluids, namely electrolyte solutions without digestive enzymes, to investigate the actual amount of proteins potentially accessible to hydrolysis during enzymatic digestion. It deserves to be noticed that the use of such electrolyte solutions and the long incubations with drastic pH change might induce a partial chemical hydrolysis of proteins; however, the actual occurrence of this side effect has been assessed by electrophoresis analysis presented in the following section. 

**Figure 1:**
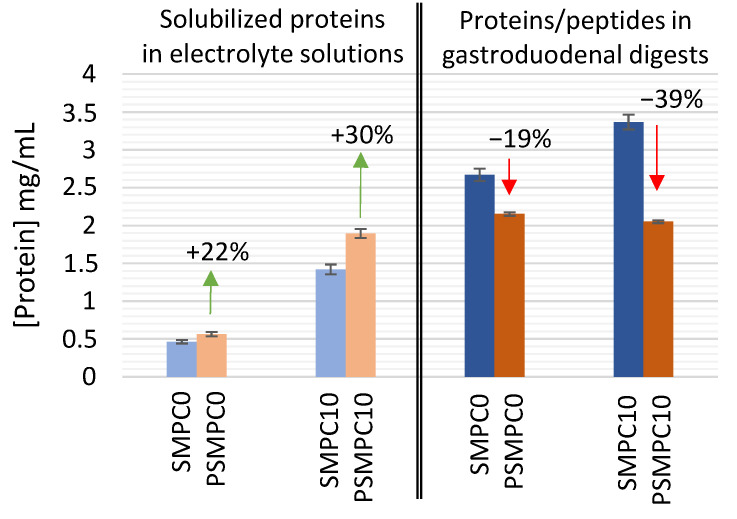
Absolute protein concentration measured by Bradford Assay in undigested (solubilized proteins in electrolyte solutions) and digested raw (SMPC0 and SMPC10) and autoclaved (PSMPC0 and PSMPC10) model sausages with and without the addition of 10% milk protein concentrate (MPC). Mean values of three independent samples have been calculated, and standard deviation is reported as error bars in the histograms.

An estimate of the amount of raw and processed (autoclaved) proteins in sausages during GD digestion was obtained. In order to keep the investigation time and cost effective, only the endpoint of the GD phase was characterized, even though monitoring several intermediate time points could have provided a further added-value to the understanding of the fate of milk proteins. As for the simulation of the physiological conditions, model sausages were subjected to the digestion procedure, where all the digestive enzymes were added. Undigested and GD-digested samples of model sausages (SMPC0, SMPC10, PSMPC0, PSMPC10) were characterized in terms of protein concentration, providing information on their solubility and enzymatic digestibility as affected by autoclaving. Results are displayed in Figure 1, as absolute protein concentration of the aforementioned samples. In order to investigate the statistical significance of the differences observed between each raw sample (SMPC0 undigested, SMPC10 undigested, SMPC0 digested, SMPC10 digested) and its relevant processed counterpart (PSMPC0 undigested, PSMPC10 undigested, PSMPC0 digested, PSMPC10 digested) an unpaired Student *t*-test with two-tailed distribution was performed at 5% significance level. Before applying the *t*-test, the equality of variances of the two independent groups was proved by an F-test, at 5% significance level. All *t*-tests confirmed that the averaged protein concentration measured by colorimetric assay for each autoclaved sample was significantly different from the relevant unprocessed sample. 

In particular, focusing on the undigested set of samples (first four columns of Figure 1), it was observed that the autoclave-based treatment increased the protein solubility by 22% in milk-free model sausages (SMPC0 vs. PSMPC0), and up to 30% in model sausages fortified with MPC (SMPC10 vs. PSMPC10). This experimental evidence suggested that the exogenous milk proteins were even more susceptible to the technological process than the meat proteins themselves. It could be hypothesized that the combination of pressure and temperature, typical of autoclaving, promotes a displacement of matrix components, leading to a modification of protein status toward a more soluble form in the processed matrix [33,34].

By performing an analogous comparison on GD-digested samples, an opposite trend was observed (last four columns of Figure 1). The processed and digested model sausages displayed an absolute protein concentration lower than their relevant unprocessed counterparts. A likely explanation of this trend might consist in an increased digestibility of the solubilized protein, accounted for by the autoclaving process itself. Indeed, hydrolyzed peptides resulting from GD digestion cannot be detected by Bradford colorimetric assay (<3 kDa), thus the reduction in absolute protein concentration of processed samples can be assumed as an indirect assessment of the higher degree of protein hydrolysis in processed sausages during GD digestion. Moreover, in this case, the autoclaving effect appeared to be particularly relevant in model sausages spiked with 10% of MPC (39% decrease in protein concentration, compared to the 19% decrease recorded for milk-free sausages), likely due to a higher susceptibility of milk proteins to the modifications induced by the technological process. It was already reported that temperatures between 100 and 120 °C could lead to the permanent aggregation of milk proteins with covalent and hydrophobic interactions causing a higher susceptibility for peptic hydrolysis [33]. Heat treatment may also lead to the occurrence of Maillard reactions, which induce radical formation with subsequent selective attack on the protein backbone [34]. Therefore, it can be assumed that autoclaving may facilitate the fragmentation of caseins, explaining the increase on protein solubility and digestibility after the heating process [35].

Lastly, Figure 1 displayed a significant increase in the absolute protein concentration of the GD-digested samples compared to the relevant undigested ones. Two likely explanations of this trend can be envisaged. First, the digested samples contain a very high amount of digestive enzymes intentionally added for the prescribed protocol and that cannot be distinguished in this kind of colorimetric assay from the proteins of interest. Second, the occurrence of the proteolytic process during the different incubation phases (gastric and duodenal) might promote a wider protein transfer from the solid food matrix to the aqueous phase of the digestive fluids.

#### 3.1.2. Characterization of the Electrophoretic Profile of Model Sausages Samples upon Technological Treatment

**Figure 2:**
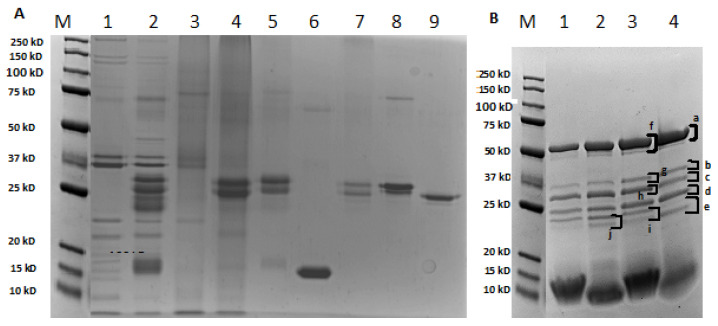
SDS-PAGE run in denaturing conditions comparing protein profiles of undigested (**A**) and digested (**B**) raw and autoclaved sausages. Legend: lane 1, SMPC0; lane 2, SMPC10; lane 3, PSMPC0; lane 4, PSMPC10; 5, MPC; 6, β-LG; 7, total caseins; 8, α-casein; 9, β-casein; a–j, selected bands for in-gel digestion and LC-MS/MS based identification. M, Precision Plus Protein™ All Blue Standard (10–250 kDa, Bio-Rad Laboratories, Segrate, Milan, Italy).

Protein fractions of raw and autoclaved model sausages were analyzed by SDS-PAGE in order to study the protein stability upon heat treatment, before and after GD digestion. Results are shown in Figure 2A,B. First, the detection of numerous protein bands at different molecular weights in both milk-free sausages (SMPC0) and sausages spiked with 10% MPC (SMPC10) allowed to confirm that the long incubation steps with electrolyte solutions (Figure 2A, lane 1 and 2) did not activate a significant chemical hydrolysis of the food proteins. A weak band was highlighted at the front of the gel, which was not related with milk proteins, since it kept the same intensity in both SMPC0 and SMPC10 samples.

As for the whole-protein profile in undigested model sausages (Figure 2A), the addition of MPC (lane 5) to the sausage produces a different protein profile (lane 2) compared to that for milk-free sausage (lane 1), with new detectable bands occurring in the ranges 25–31 and 15–20 kDa. The latter bands can be assigned to caseins and to whey proteins, respectively by comparison with the electrophoretic profile of different standards run on the same gel. Indeed, the bands at 25–31 kDa coincide with those obtained in lanes 7, 8, and 9 corresponding to total caseins and purified α- and β-caseins, respectively, while bands between 15 and 20 kDa can correspond to β-LG as confirmed by lane 6 loaded with the purified protein. 

Noteworthily, very weak protein bands appear in the milk-free sausage profile after the autoclave-based treatment (Figure 2A, lane 3) that where reduced in number and intensity compared to that in the raw sample (lane 1). The latter represents a clear proof of the effect of autoclaving treatment on the stability of endogenous proteins of sausage (turkey and pork), with almost complete degradation of most of the bands. As for sausage with 10% of MPC (Figure 2A, lane 4), bands around 25 kDa, assigned to caseins group, are still strongly visible after autoclaving, while those corresponding to whey proteins (15–20 kDa) where significantly reduced and/or degraded. These results are concordant with the principle that whey proteins are largely affected by heat treatment, with the subsequent denaturation of their tertiary and quaternary structures [36,37]. Additionally, there is also the possibility of increased hydration of protein molecules and/or irreversible aggregation of whey proteins and whey proteins with caseins [33,36,37,38]. In this way, whey protein aggregates formed after autoclaving may not be visible because of their high MW (>250 kDa), which hindered their entrance into the gel [38]. These facts may be caused by the occurrence of Maillard reactions between milk proteins and lactose or protein binding to fat globule membrane present in MPC after thermal treatment [38]. Accordingly, Bu et al. [37] reported a significant decrease in β-LG allergenicity when temperatures above 90 °C were applied. α-LA has a similar behavior, but with a greater decrease, only at 120 °C during 20 min.

Electrophoretic profiles of both raw and processed sausages after the GD digestion (Figure 2B, lane 1–4), deeply changed in comparison with the respective undigested profiles (Figure 2A, lane 1–4). As seen from Figure 2B, all model sausages (lane 1–4) are characterized by one intense band at approximately 50 kDa and four defined bands with MW between 23 and 37 kDa. In addition, one smeared band was detectable in the region of 10 kDa, whose intensity decreased with autoclaving, both in sausages without and with MPC addition (Figure 2B, lanes 2 and 3). In order to deepen the information about the fate of autoclaved proteins during digestion, some selected bands (a–j, Figure 2B) were in-gel digested, and the resulting tryptic peptides were analyzed by LC-MS/MS for protein identification. Bioinformatic investigation revealed that all the analyzed bands correspond to a mixture of digestive enzymes. This clearly suggests that during the different hydrolytic phases of the GD process most of the soluble proteins in physiological digestive fluids were almost completely digested by proteolytic enzymes, as also demonstrated by the intense smeared signal banding between 15 and 10 kDa, likely produced by co-migrating peptides arisen from protein digestion. Noteworthily, such a low-molecular-weight smeared band in the two lanes of Figure 2B related to 10% MPC–added sausages (lane 2: digested SMPC10, lane 4: digested PSMPC10) appeared to be more intense toward 10 kDa, suggesting that milk proteins are quite susceptible to in vitro GD digestion. In addition, the autoclave-based process further promoted the proteolytic degradation, resulting in a lighter band of digested PSMPC10 than digested SMPC10. These results are in accordance with the decrease in protein concentration measured by colorimetric assay presented in Figure 1 (see Section 3.1.1).

#### 3.1.3. LC-MS/MS Analysis of Sausage Material upon Gastroduodenal Digestion

In order to have additional information about the digestibility of milk proteins in model sausages, and, in particular, the putative enhancement effect of autoclaving on protein hydrolysis, the digested SMPC10 and PSMPC10 samples were characterized by LC-MS/MS analysis. MS data were processed via Proteome Discoverer software for protein/peptide sequence identification by searching against a customized database including the three main expected taxonomies (*Sus scrofa*, *Meleagris gallopavo,* and *Bos taurus* species), as well as sequences of the digestive enzymes used for simulating GD digestion. Individual proteins identified are summarized in Table 2.

In general, all proteins derived from the meat used in sausage preparation were fully degraded upon GD digestion, indeed no accession belonging to turkey or pork species was detected within the sensitivity limit of this analytical approach. In the case of milk proteins, some casein peptides appeared to survive the GD digestion, in fact in both digested SMPC10 and PSMPC10 samples, specific peptides belonging to β-, κ-, and αS_1_-caseins (Table 2) were found. 

**Figure 3:**
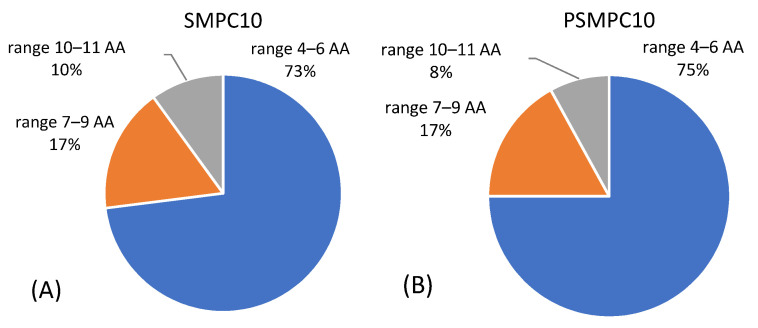
Overview of peptide distribution across three groups differing for sequence length (ranges: 4–6 amino acid (AA), 7–8 AA, 9–11 AA) and detected in digested (**A**) SMPC10 and (**B**) PSMPC10 model sausages.

In light of this, the higher number of peptides identified for β-casein in both sausage samples highlights that this protein was digested to a lesser extent than the other caseins, for which very few peptides were retrieved by the software (Table 2). Although exhibiting the same protein composition, a distinct number of peptides was observed for each protein in raw and autoclaved-digested sausages. Specifically, a lower number of peptides was recognized in processed sausages for β-casein and k-casein, suggesting that the chemical/structural changes induced by autoclaving and food matrix could promote enzymatic activity, thus, increasing the digestion rate of these proteins. On the contrary, no significant difference was displayed for αS_1_-casein in raw and autoclaved mixtures for which two peptides were identified in both cases. The extensive fragmentation of proteins induced by digestion and increased by pressure/thermal treatment was also demonstrated by the average length of peptide sequences identified in digested sausages before and after autoclaving for each casein. In Figure 3, all peptides retrieved by the software for SMPC10 and PSMPC10 digested sausages were grouped according to their length (in three windows, namely 4–6 amino acid (AA), 7–8 AA, and 9–11 AA), and the number of total peptides were detected in each model sausage. 

As seen from the graph, the majority of peptides is made up of a short amino acid sequence (4–6 AA) in digests of raw and autoclaved sausages with MPC. In particular, a slightly higher number of peptides in this range was recorded for autoclaved sausages compared to raw ones (Figure 3). In line with this, also the number of longer peptides comprised in the range of 10–11 AA recorded in raw sausages with MPC was higher than those recorded in autoclaved ones. As expected, and already described in Section 3.1.1, harsh heat treatments (such as autoclave) can promote protein fragmentation and facilitate digestibility, resulting in a higher number of short peptides, principally from caseins [34,39]. It is also feasible to assume that due to the extensive fragmentation induced by this treatment, peptides shorter than 4 AA were generated, which could not be identified by the software.

### 3.2. Assessment of IgE-Binding Capacity of Autoclaved Samples

**Figure 4:**
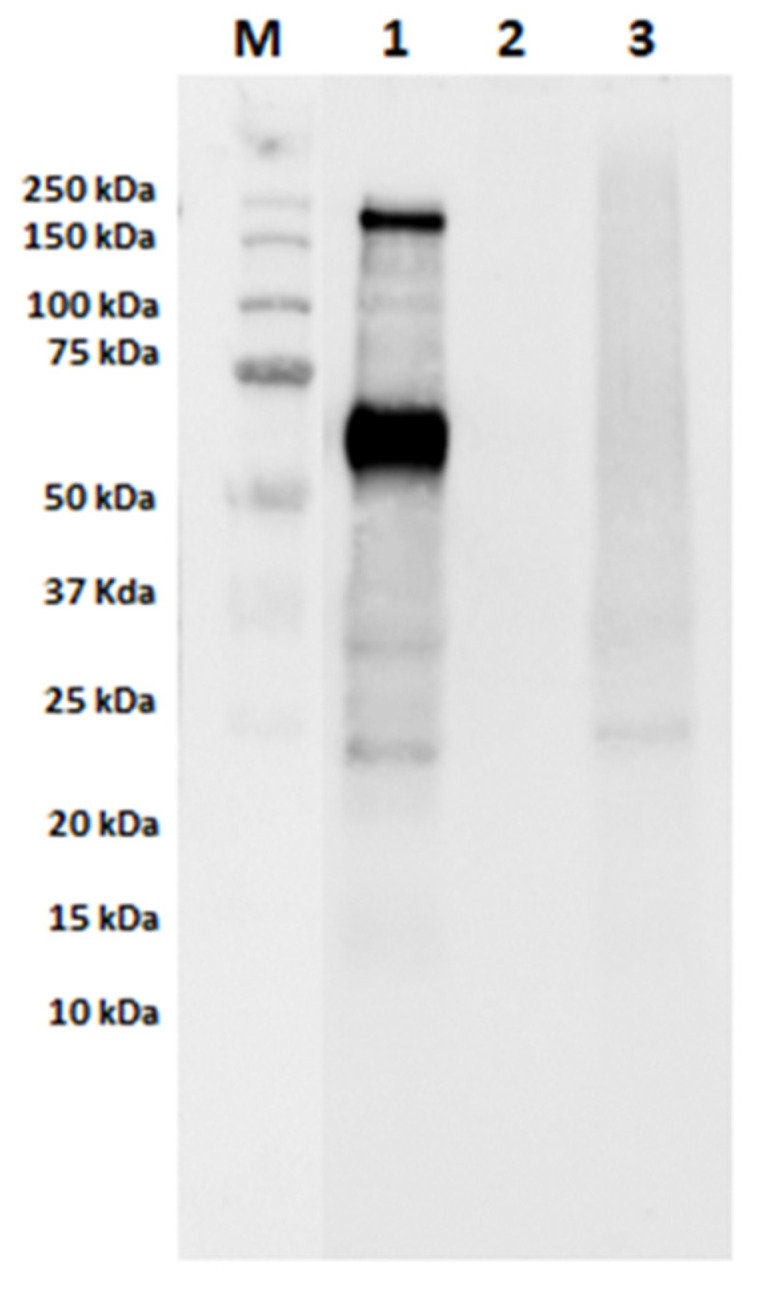
Immunoblot of undigested raw and autoclaved model sausages. Legend: lane 1, SMPC10; lane 2, PSMPC0; lane 3, PSMPC10; M, Precision Plus Protein™ All Blue Standard (10–250 kDa, Bio-Rad Laboratories, Segrate, Milan, Italy). The immunoblot was carried out on a pool of sera of patients allergic to cow’s milk.

In order to evaluate the effects of autoclaving and GD digestion on the final IgE-binding capacity of milk proteins in model sausages, immunoblotting experiments using sera of cow’s-milk-allergic patients were performed. Specifically, undigested raw and autoclaved model sausages containing MPC along with their digested counterparts and their respective controls (milk-free autoclaved samples) were analyzed. In Figure 4, raw model sausage containing MPC showed two main reactive bands at approximately 200 and 60 kDa, the latter putatively assigned to BSA (undigested SMPC10 sample; Figure 4, lane 1). Notwithstanding the high reactivity to caseins assayed in the serum samples (see Table 1), only a weak IgE-reactivity was observed in bands between 20 and 30 kDa, which correspond to caseins. No bands corresponding to the milk allergens ß-LG and α-LA were recognized by the pool of sera in any undigested model sausage. In the autoclaved model sausage, a complete loss of IgE reactivity of BSA and the 200 kDa band could be observed, suggesting that this thermal treatment greatly reduces the IgE-binding capacity of these proteins (PSMPC10; Figure 4, lane 3). 

As for caseins, the final IgE-binding capacity seems to be reduced, but the two weak signals previously assigned are still visible in autoclaved sample (Figure 4, lane 3). Our findings are in accordance with results published by other authors, suggesting that caseins are heat-stable proteins with persistent IgE-binding capacity [35]. As already reported by Bloom et al. [40], sera from milk-allergic subjects continued IgE-reactive to caseins, even following harsh heat treatment (60 min at 95 °C). In addition, our findings showed a clear loss of BSA antigenicity after the autoclaving treatment, due to the harsh conditions applied during this treatment combining elevated temperatures (121 °C) with high pressure (1 bar). As already stated, besides total protein, MPCs contain fat and lactose, which may react with milk proteins by heat treatment, possibly affecting their IgE-binding capacity. In this regard, Xu et al. [38] found that, under heating condition and in the presence of a certain amount of lactose, the Maillard reaction may induce the loss of linear epitopes of milk allergens, thus reducing their final antigenicity. Lower solubility caused by the development of high-molecular-weight aggregates between whey proteins and caseins after thermal treatment may also lead to a reduction of their antigenicity [37]. The involvement of the food matrix in the reduction of IgE-binding capacity needs also to be considered since, during food processing, the formation of complexes between food matrix and milk proteins could occur, thus masking some allergenic epitopes with a consequent decrease of casein antigenicity as reported by Bavaro et al. [17]. In the light of this, the contribution of the meat matrix to the reduction of IgE binding with milk proteins should not be excluded.

Raw and autoclaved sausages submitted to GD digestion were evaluated by immunoblotting experiments with the sera of milk-allergic patients as well. As expected, due to the extensive digestion of the milk proteins along the GD digestion (see Bradford assay and SDS-PAGE experiments, Section 3.1.1 and Section 3.1.2), no IgE-binding capacity was observed for all the tested models (data not shown). In order to further confirm the absence of any residual immunogenicity in digested model sausages, the peptide sequences identified by proteomic analysis in raw and autoclaved models with the addition of MPC (SMPC10 and PSMPC10) submitted to GD digestion were screened into the IEDB database for retrieving surviving milk linear epitopes for the *Homo sapiens* host. By searching exact matches with known epitopes, no hit was disclosed in the short list of sequences detected as GD-resistant peptides, confirming the loss of IgE-binding capacity after GD digestion. 

Trying to deepen the investigation, the peptide list was browsed again by activating the “substring” option, namely by assessing whether the detected peptides may represent shorter fragments of known epitopes, and in this case some hits were found (itemized in Table S1), for digested SMPC10 or PSMPC10 samples. Twelve peptides containing partial epitopes were detected in the raw model sausage SPMC10, which appeared to be highly susceptible to the technological treatment, and they were completely degraded in the relevant autoclaved sample PSMPC10 (not detected). In addition, 11 peptides belonging to β-casein were found both in SMPC10 and PSMPC10, highlighting their partial resistance not only to the GD digestion but also to the thermal/pressure treatment. Finally, seven peptides including new epitopes fragments were reported for PSMPC10, likely deriving from different proteolytic pathways originated in β-casein digestion on account of its preliminary treatment with the autoclave. Concerning k-casein, the majority of peptides were conserved after autoclaving, and similar results were obtained for α-casein. In conclusion, the few milk peptides detected at the end of GD digestion in model sausages did not encrypt known full-length epitopes but presented only a partial match with epitope fragments. The actual IgE reactivity of these fragments is unknown and likely lost; indeed, shorter sequences should have drastically impaired their affinity for IgE-binding sites. This bioinformatic characterization can confirm the previous results from immunoblotting experiments on GD digests as absence of the IgE-binding activity. In addition, the detection of some differences in the peptide sequences of GD-resistant peptides of PSMPC10 vs. SMPC10 suggested that the autoclave-based process has an important role in providing a further degradation of proteins preliminary to the GD digestion itself. 

## 4. Conclusions

In summary, the autoclaving treatment here investigated on model sausages fortified with milk proteins was proved to positively affect protein solubility in electrolytic solutions simulating digestive fluids promoting their accessibility to the proteolytic enzymes involved in the human GD digestion process. The combined effect of the thermal/pressure-based degradation by autoclaving with the enzymatic hydrolysis occurring in GD digestion simulated in vitro allows the significant fragmentation of milk proteins down to low-molecular-weight peptides (≤10 kDa). Analysis by LC-MS/MS demonstrated that these peptides correspond to caseins fragments (αS1, β-, and κ-caseins), with β-casein being the most resistant after digestion. On the contrary, whey proteins were completely degraded by digestion. 

Immunoblotting experiments carried out with sera from milk-allergic individuals on undigested model sausages confirmed that the technological treatment deeply affects the IgE-binding activity of the milk proteins. In addition, after simulated GD digestion, no IgE-reactivity was observed for any milk proteins, and this evidence was also confirmed by LC-MS/MS analysis and bioinformatics search of intact known epitopes. The in silico analysis by the IEDB portal proved the absence of intact IgE-reactive epitopes of milk proteins and the presence of only a partial match with epitopic sequences that partially survived the proteolytic process. 

These findings can provide a useful contribution for allergists and the food industry since autoclaving proved to produce a great effect on the immunoreactivity of milk proteins used as technological aids in meat products. This fact highlights the importance of the use of food processing techniques for the development of hypoallergenic formulas. 

## Figures and Tables

**Table 1:** Overview of the individual serum reactivity and clinical symptoms displayed by the allergic children involved in this study.

Sera	Total IgE (kU/L)	sIgE to Cow’s Milk (kUA/L)	sIgE to Caseins (kUA/L)	sIgE to α-Lactoalbumin (kUA/L)	sIgE to β-Lactoglobulin (kUA/L)	Allergic Reaction Displayed
1	66	26.8	6.32	8.91	3.19	anaphylaxis
2	227	19	17.5	3.49	0.19	cough and rhinitis
3	4798	38.1	20.7	17.5	16.5	anaphylaxis
4	88	22.7	21.1	3.06	0.59	vomit
5	1629	24.1	28.6	1.62	6.05	anaphylaxis
6	439	13.9	6.24	3.79	0.27	vomit
7	115	>100	>100	20.4	5.08	anaphylaxis

**Table 2:** Summary of proteins identified by LC-MS/MS analysis and software-based identification in raw (SMPC10) and autoclaved (PSMPC10) model sausages after gastroduodenal digestion. Please refer to Section 2.7.3 for full details about the sequence identification procedure.

Sample	Accession	Description	Coverage	Peptides (Unique)	PSMs ^a^	Score	MW ^b^ [kDa]
**SMC10-Dig**	P02666	β-casein OS ^c^ = *Bos taurus*	58.04	21 (14)	660	106.64	25.1
	P02668	k-casein OS = *Bos taurus*	32.11	5 (1)	55	5.42	21.3
	P02662	αS_1_-casein OS = *Bos taurus*	41.59	2 (2)	103	4.23	24.5
**PSMC10-Dig**	P02666	β-casein OS = *Bos taurus*	62.95	18 (11)	814	108.87	25.1
	P02668	k-casein OS = *Bos taurus*	34.21	4 (2)	59	3.84	21.3
	P02662	αS_1_-casein OS = *Bos taurus*	59.81	2 (2)	164	2.63	24.5

^a^ PSMs, peptide spectrum matches; ^b^ MW, molecular weight; ^c^ OS, organism source.

## Data Availability

The data presented in this study are available in the article and the supplementary materials.

## Supplementary Materials

The following are available online at https://www.mdpi.com/2072-6643/13/3/931/s1, Table S1: Overview of peptides retrieved by the Proteome Discoverer software for each protein by analyzing raw (SMPC10) and autoclaved (PSMPC10) gastroduodenal-digested sausages. Immune Epitope database (IEDB) results by activating “substring” filtering were also reported. The symbol “x” marks samples where individual peptides were found, while “/” stands for “not found”.
