# Supplementary material for: Milk Ingredients in Meat Products: Can Autoclaving and In Vitro Gastroduodenal Digestion Mitigate Their IgE-Binding Capacity?
Source: Nutrients. 2021 Mar 13;13(3):931. doi: 10.3390/nu13030931 (PMC8000631; doi:10.3390/nu13030931)
Supplement: Supplementary file 1 [file nutrients-13-00931-s001.pdf]

Table S1. Overview of peptides retrieved by the Proteome Discoverer software for each protein by analyzing raw (SMPC10) and autoclaved (PSMPC10) gastro-duodenal digested sausages. IEDB results by activating "substring" filtering were also reported. Symbol "x" marks sample where individual peptide was found, while "/" stays for "not found".

Protein (accession)	Peptide sequence	SMPC10 digest	PSMPC10 digest	IEDB Epitope Antigens	ID Epitope (substring)
**ß-casein OS=*Bos taurus* (P02666)**	PVVVPPFLQPE	x	x	Bos d 11 *Bos taurus* (bovine)	115393, 115420, 115460, 115907
	HQPHQPLPPT	x	x	/	/
	HIPLP	x	x	/	/
	PVVVPP	x	x	Bos d 11 *Bos taurus* (bovine)	115393, 115420, 115460, 115907, 115931
	PVIGPV	x	x	/	/
	PFPGPI	x	x	Bos d 11 *Bos taurus* (bovine)	59576, 78152, 115246, 115883, 115973, 116025, 229825, 229827, 229828
				Bubalus bubalis (domestic water buffalo) protein	229826
	IPIP	x	x	/	/
	YPVEP	x	x	Bos d 11 *Bos taurus* (bovine)	30533,115216,115280,115742, 115839, 115865, 229605, 229632, 229673, 229674, 229741, 229759, 229760, 229762, 229767
	VYPFPGPI	x	x	Bos d 11 *Bos taurus* (bovine)	59576, 78152, 115246, 115973, 116025, 229825, 229826, 229827, 229828
	PVVVPPF	x	x	Bos d 11 *Bos taurus* (bovine)	115393,115420, 115460, 115907,115931
				ß-casein *Capra hircus* (domestic goat)	227136
	VVPPF	x	x	Bos d 11 *Bos taurus* (bovine)	115393, 115420, 115460, 115554, 115907, 115931
	EMPFPK	x		Bos d 11 *Bos taurus* (bovine)	30533, 115216, 115280, 115807, 115823, 229605, 229632, 229673, 229674, 229762, 229767
	PQNIPPL	x		Bos d 11 *Bos taurus* (bovine)	115899, 115969
	LNVPGE	x		Bos d 11 *Bos taurus* (bovine)	53557, 115219, 115466, 115725, 115842
	MAPK	x		Bos d 11 *Bos taurus* (bovine)	115216
	MHQPHQPLPPT	x		/	/
	RGPFPL	x		/	/
	RGPFP	x		Bos d 11 *Bos taurus* (bovine)	115251, 115298, 115763, 116016
	PFPK	x		Bos d 11 *Bos taurus* (bovine)	30533, 115216, 115280, 115807, 115823, 115865
	MFPPQ	x		Bos d 11 *Bos taurus* (bovine)	38630, 51872, 78287, 115904, 116019
				Capra hircus (domestic goat)	227654

	MPFPK	x		Bos d 11 *Bos taurus* (bovine)	30533, 115216, 115280, 115807, 115823, 115865, 229605, 229632, 229673, 229674, 229741, 229762, 229767
	EMPFP	x		Bos d 11 *Bos taurus* (bovine)	30533, 115216, 115280, 115807, 115823, 229605, 229632, 229673, 229674, 229762, 229767
	QPHQPLPPT	x		/	/
	PLPPT		x	Bos d 11 *Bos taurus* (bovine)	51872, 115923,
	MFPPQ		x	Bos d 11 *Bos taurus* (bovine)	38630, 51872, 7828, 115904, 116019
				ß-casein *Capra hircus* (domestic goat)	227654
	PFPK		x	Bos d 11 *Bos taurus* (bovine)	30533, 115216, 115280, 115807, 115823, 115865
	PPFLQPE		x	Bos d 11 *Bos taurus* (bovine)	115393, 115420, 115460, 115554, 115895
	EMPFPK		x	Bos d 11 *Bos taurus* (bovine)	30533, 115216, 115280, 115807, 115823, 229605, 229632, 229673, 229674, 229762, 229767
	RGPFPI		x	Bos d 11 *Bos taurus* (bovine)	115251, 115763
	YPVEPF		x	Bos d 11 *Bos taurus* (bovine)	30533, 115216, 115280, 115742, 115839, 229605, 229632, 229673, 229674, 229741, 229759, 229760, 229762, 229767
**κ-casein OS=*Bos taurus* (P02668)**	MAIPPK	x	x	Bos d 12 *Bos taurus* (bovine)	115468, 47842, 115281, 115369, 115379, 115517, 115741, 115853, 161638
	IPYP	x	x	/	/
	HPHP	x	x	Bos d 12 *Bos taurus* (bovine)	54078, 115195, 115468, 54079, 115175, 115450, 115517, 115863, 116008
	HPHPH	x		Bos d 12 *Bos taurus* (bovine)	54078, 115195, 115468, 54079, 115175, 115450, 115517, 115863, 116008
	IPPK	x		Bos d 12 *Bos taurus* (bovine)	115468, 47842, 115281, 115369, 115379, 115517, 115678, 115741, 115853, 161638,
	FIPYP		x	/	/
**αS$_1$-casein OS=*Bos taurus* (P02662)**	HQGLPQ	x	x	Bos d 9 *Bos taurus* (bovine)	115282, 31145, 109844, 115311, 190448, 229677
	VAPFPEV	x	x	Bos d 9 *Bos taurus* (bovine)	38207, 43705, 44794, 15930, 15931, 67707, 67708, 67709, 69660, 109844, 110049, 115081, 115396, 115436, 115467, 115531, 190478
				Other *Capra hircus* (goats) protein	229748
